# Tracing Obesity From Parents to Adult Offspring: The Tromsø Study 1994–2016

**DOI:** 10.1155/jobe/8834694

**Published:** 2025-10-11

**Authors:** Mari Mikkelsen, Tom Wilsgaard, Sameline Grimsgaard, Bjarne K. Jacobsen, Laila A. Hopstock

**Affiliations:** ^1^Department of Community Medicine, UiT the Arctic University of Norway, Tromsø, Norway; ^2^Centre for Sami Health Research, Department of Community Medicine, UiT the Arctic University of Norway, Tromsø, Norway; ^3^Department of Health and Care Sciences, UiT the Arctic University of Norway, Tromsø, Norway

**Keywords:** adult offspring, body mass index, family, intergenerational, obesity

## Abstract

**Objective:**

The combination of genetic and environmental contributors to obesity can be studied through intergenerational associations as previously shown in studies of parents and their children and adolescents. Few studies have investigated this in adulthood. This study aims to explore sex-specific associations in body mass index (BMI) and obesity status between parents and their adult offspring.

**Methods:**

We used cross-sectional data from two surveys in the population-based Tromsø Study. Individuals participating in the seventh (Tromsø7 2015–2016) survey were linked to their parents in the fourth (Tromsø4 1994–1995) survey. Data were analyzed using linear mixed models and generalized estimating equations adjusting for sibling clusters, parents' age and education level, and offspring's sex, age, education, and physical activity level. The analytical sample included 2068 parent-offspring trios, both parents and offspring aged 40–59 years.

**Results:**

Results showed strong associations between parents' and adult offspring's BMI and obesity status, which remained strong after adjustments. Having two parents with obesity (compared to normal weight) showed a 3 times higher risk of obesity in the offspring. Mother-daughter relationships tended to be stronger than mother-son relationships.

**Conclusion:**

Our study adds to previous studies of familial transmission of obesity from parents to their young children and adolescents, confirming these associations persist into middle age.

**Summary**

• What is already known?
◦ Previous studies show positive associations between parents' and their children's BMI and obesity status in childhood and further into adolescence.
• What does this study add?
◦ Our findings show that the association between parents' and offspring's BMI and obesity status is present also in middle age.
• How might these results change the direction of research or the focus of clinical practice?
◦ Our findings highlight the importance of early intervention and prevention strategies to mitigate the long-term effects of obesity passed on in families.

## 1. Introduction

The global obesity prevalence has more than doubled since 1990 [1], and in 2022 almost 60% of adults in Europe had overweight or obesity [2]—conditions increasing the risk of noncommunicable diseases and mortality [1]. Genetic factors contribute to an individual's predisposition to obesity [3], increasing the risk of developing obesity when exposed to an obesogenic environment, indicating a gene-environment interaction [4, 5]. Increasing body mass index (BMI) is seen among both those with and without a genetic risk profile, indicating a considerable contribution from environmental factors [6]. The combination of genetic and environmental factors can be studied through intergenerational associations. Meta-analysis shows strong associations between parents' and their children's obesity status [7]. A recent meta-analysis studying adult offsprings (of all ages) also found positive associations [8]. Among studies in adult offsprings, some have found positive associations between parents' and adult offspring's obesity status up to young adulthood [9, 10], early age (44–45 years) [11], and older age [12–15]. However, some of these studies relied on self-reported weight and height, potentially causing misclassification bias [12, 16], some did not include maternal and paternal BMI in the same analysis [15], and some did not separate young and middle-aged or older adults [14]. Studies suggest that the intergenerational transmission of overweight and obesity may depend on the parents' and offspring's sex [17, 18], although results are conflicting [19].

Thus, although the link between the weight status of parents and their children is well documented, knowledge of this relationship during middle age remains limited. Therefore, this study aims to explore sex-specific associations between the BMI and obesity status of parents and their adult offspring in an ongoing population study.

## 2. Methods

### 2.1. Study Population

The Tromsø Study [20–22] is an ongoing population-based health study conducted in the municipality of Tromsø, Norway, with 7 surveys during 1974–2016 (Tromsø1-Tromsø7) including > 45,000 participants attending one or more surveys. Data collection comprises clinical examinations, biological sampling, questionnaires, and interviews. The number of invited participants varies between the surveys, mainly depending on available funding.

### 2.2. Study Sample

The current study includes data from the fourth (Tromsø4) and the seventh (Tromsø7) survey of the Tromsø Study. In Tromsø4 (1994–1995), all inhabitants in Tromsø municipality aged 25 years and older (i.e., born before 1970) were invited (*N* = 37,558) [20]. In total, 27,158 (77% attendance, corrected for individuals who moved or died before the survey) women and men aged 25–97 years participated. An information brochure and a short questionnaire followed the invitation letter, and a more extensive questionnaire was handed out upon attendance. A subsample of 7965 women and men participated in extended clinical examinations including measurements of waist circumference. In Tromsø7 (2015–2016), all inhabitants aged 40 years and older (i.e., born before 1975) were invited (*N* = 32,591) [22]. A total of 21,083 (65% attendance) women and men aged 40–99 years participated. An information brochure and a short questionnaire followed the invitation letter, as well as log-in details for online completion of extensive questionnaires. A food frequency questionnaire (FFQ) was handed out upon attendance.

**Figure 1:**
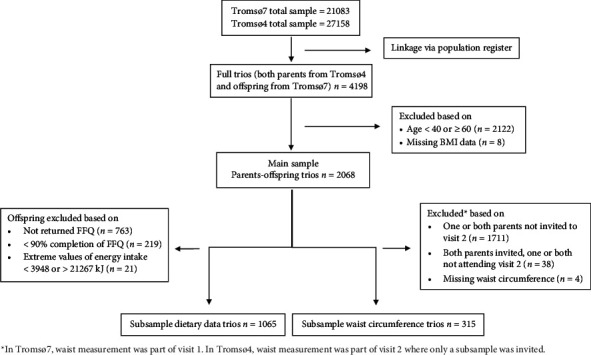
Flowchart of study sample.

With family linkage via the National Population Register [23], we linked participants aged 40–59 years when attending Tromsø7 to their parents aged 40–59 years when participating in Tromsø4. We selected middle-aged adults for our analysis to minimize the risk of bias associated with age-related diseases that could potentially influence BMI in older age groups. To calculate parent-specific associations with reduced bias from missing parent data, we included full parent-offspring trios (mother, father, and offspring). We excluded participants outside the selected age range (*n* = 2122) and with missing data on BMI (*n* = 8), resulting in a sample of 2068 parent-offspring trios for the main analyses. Further, a sample of 1065 parent-offspring trios was available for subanalysis including valid dietary data for the offspring. Moreover, a sample of 315 parent-offspring trios was available for subanalysis including waist circumference data in parents and the offspring. Inclusion of participants is presented in Figure 1.

### 2.3. Ethics and Privacy

The study was approved by the Regional Committee for Medical and Health Research Ethics North (reference 315,696). The processing of personal data was approved by Sikt (ref. 818,086). Data access was approved by the Tromsø Study Data and Publication Committee. Participants in both surveys gave written consent.

### 2.4. Measurements and Variables

Age in years was included in the analyses as a continuous variable. Weight and height were measured in light clothing and no shoes. Waist circumference was measured to the nearest centimeter (cm) at the umbilical level using a measurement tape. Trained personnel performed all measurements using standard procedures. BMI was calculated as the individual's weight divided by the square of their height (kg/m^2^). BMI categories were defined in accordance with WHO [1] (normal weight < 25 kg/m^2^, overweight 25–29.9 kg/m^2^, or obesity ≥ 30 kg/m^2^). The parents' combined individual BMI categories were merged into 5 new categories: both parents had normal weight, one or both parents were overweight (none of them had obesity), mother (but not father) had obesity, father (but not mother) had obesity, both parents had obesity.

Education level in parents and offspring was measured by the question: “What is the highest level of education you have completed?” defined as primary (primary school/folk high school), secondary (high school), tertiary short (college/university less than 4 years), or tertiary long (college university/university 4 or more years). Information about the offspring also included data on physical activity and diet. Physical activity in leisure time level was measured by the Saltin–Grimby Physical Activity Level Scale [24]: “Describe your exercise and physical exertion in leisure time” defined as sedentary (reading, watching TV, or other sedentary activity), low (walking, cycling, or other forms of exercise at least 4 h a week), moderate (participation in recreational sports, heavy gardening, etc. at least 4 h a week), or vigorous (participation in hard training or sports competitions regularly several times a week). The question referred to averages during the last year. Data from a previously validated FFQ [25] (261 questions on frequency and amount of food intake) was computed into daily energy, food, and nutrient intake as described in detail elsewhere [26]. In accordance with Lundblad et al. [26], participants answering less than 90% of the FFQ items (*n* = 982) and with extreme values for total energy intake (< 1 and > 99 percentile) (*n* = 21) were excluded. Total energy intake in megajoule (MJ) was included in subanalyses.

### 2.5. Statistical Analyses

We used linear mixed models to assess the association between adult offspring's BMI as a dependent variable and parents' BMI as exposure variables. Parents' BMI was standardized with standard deviations (SD) calculated separately for mothers and fathers. Mother's pseudonymized ID was included as a random effect to account for sibling clusters. The associations were estimated by adjusting for parents' and offspring's age (model 1) and additionally by parents' and offspring's education and offspring's physical activity (model 2). Education and physical activity were included in the analyses as continuous variables (coded 1–4), which assume a linear association over four categories. This gave a better model fit with a slightly lower Akaike Information Criterion (AIC) value compared to a model where these variables were defined as categorical. Tests of interactions between mother's and father's BMI and offspring's sex were assessed by adding two-way cross-product terms to a fully adjusted model including daughters and sons together. Otherwise, analyses were stratified on offspring's sex. We did supplementary analyses studying the association between parent's and offspring's height and weight, separately, adjusting for the same covariates. For parents, height and weight were standardized with SDs calculated separately for mothers and fathers. Results are presented as regression coefficients with 95% confidence intervals (CIs). All statistical analyses were performed using STATA 18.

We used generalized estimating equations (GEE) with a Poisson distribution, log link function, and robust standard errors to examine the association between offspring's obesity status (obesity yes/no) as dependent variable and parents' combined BMI category as exposure variable. We chose an exchangeable correlation structure, assuming constant correlation between siblings. Combinations of parents' BMI categories were included as indicator variables for each category using both parents with normal weight as the reference category. Test of interaction between parents' BMI categories and offspring's sex were assessed by adding cross-product terms between offspring's sex and each indicator variable of parents' BMI category to a fully adjusted model including daughters and sons. The overall significance of the interaction terms was tested with a Wald test. Otherwise, analyses were stratified on offspring's sex. Results from sex-stratified analyses were presented as risk ratios (RRs) with 95% CIs and adjusted for the same set of covariates as in the linear mixed models.

In the analysis of subsamples, we included total energy intake for the offspring to the linear mixed model assessing the association between parents' and offspring's BMI. Further, in subsamples with measurements of waist circumference, we examined the association between parents' and offspring's waist circumference using a linear mixed model and adjusting for the same covariates as the main analyses. Due to smaller groups in the subsamples, analyses were adjusted for, rather than stratified on, sex. Mother's and father's waist circumferences were standardized with SDs calculated separately for mothers and fathers. Test of interaction between mother's waist circumference and offspring's sex were assessed by adding two-way cross-product terms to a fully adjusted model.

## 3. Results

Study sample characteristics are presented in Table 1. Among Tromsø7 participants (adult offspring), daughters had lower BMI, fewer daughters had obesity, and more daughters reported low levels of physical activity and higher education than sons. Among Tromsø4 participants (parents), fathers had higher age and BMI, and more fathers had obesity than mothers. Fewer parents had attained higher education compared to offsprings.

### 3.1. Association Between Parents' and Adult Offspring's BMI

Linear mixed models showed positive associations between parents' and offspring's BMI, which remained strong after adjustments. One SD increase in mother's BMI (4.0 kg/m^2^) was associated with 0.95 (95% CI 0.63, 1.27) and 0.62 (95% CI 0.38, 0.87) BMI units increase in daughter's and son's BMI, respectively, when adjusted for all covariates (Model 2, Table 2). No significant interaction was observed between maternal BMI and offspring's sex (*p*=0.11), or between paternal BMI and offspring's sex (*p*=0.38). One SD increase in father's BMI (3.1 kg/m^2^) was associated with 0.80 (95% CI 0.48, 1.12) and 0.68 (95% CI 0.43, 0.92) BMI units increase in daughter's and son's BMI, respectively, when adjusted for all covariates (Model 2, Table 2). The intraclass correlation coefficient (ICC) for the fully adjusted model was 0.38 and 0.31 for women and men, respectively. Offspring's education level (in daughters only) and physical activity level (daughters and sons) showed significant inverse association with BMI but had only marginal impact on the BMI association. Higher education in the mother was associated with lower BMI in sons, but adjusting for education did not affect the association between mother and son's BMI. Otherwise, parents' education was not significantly associated with offsprings' BMI. We found positive associations between parents' and offspring's height (Table S1) and weight adjusted for height (Table S2).

### 3.2. Association Between Parents' and Adult Offspring's Obesity Status

**Figure 2:**
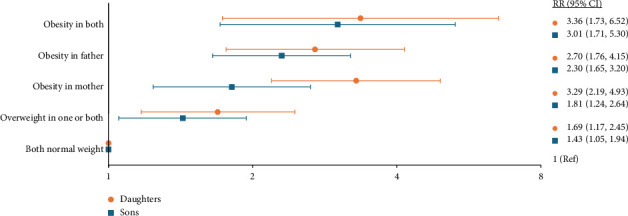
Risk ratio of obesity in offspring by parent's obesity status.

GEE analyses showed a strong association between parents' and offspring's obesity status, which remained strong after adjustments. The offspring had increased risk of obesity if one or both parents were overweight (RR 1.69 (95% CI 1.17, 2.45) in daughters and RR 1.43 (95% CI 1.05, 1.94) in sons) and a higher risk if the mother had obesity (RR 3.29 (95% CI 2.19, 4.93) in daughters and RR 1.81 (95% CI 1.24, 2.64) in sons), or if the father had obesity (RR 2.70 (95% CI 1.76, 4.15) in daughters and RR 2.30 (95% CI 1.65, 3.20) in sons), when adjusted for all covariates (Model 2, Table S3). If both parents had obesity, the offspring had more than 3 folds increased risk of obesity (RR 3.36 (95% CI 1.73, 6.52) in daughters and RR 3.01 (95% CI 1.71, 5.30) in sons). The results are illustrated in Figure 2. The overall test of interaction between BMI status in parents and offspring's sex was nonsignificant (*p*=0.18), although the risk of offspring obesity when only the mother had obesity was significantly stronger in daughters than sons (*p*=0.03). Physical activity level showed significant inverse association with offspring's obesity status in daughters and sons. Mother's education level was inversely associated with son's BMI. Adjusting for these variables, did, if anything, strengthen the association with obesity status.

RRs from GEE analysis of the association between parents' and offspring's obesity status adjusted for parents' age and education level and offspring's age, physical activity, and education level. *X*-axis shows RR values on a logarithmic scale.

RR; risk ratio, CI; confidence interval. Overweight is defined as BMI 25.0–29.9 kg/m^2^ and obesity as BMI ≥ 30.0 kg/m^2^.

### 3.3. Subsamples

#### 3.3.1. Adjusting for Offspring's Energy Intake

In a subsample of 1065 parent-offspring trios, we found that the association between parents' and offspring's BMI remained strong when adjusting for offspring's energy intake. Before including energy intake, when adjusting for all other covariates (Model 2, Table S4), one SD increase in mother's and father's BMI was associated with a 0.75 (95% CI 0.47, 1.03) and 0.78 (95% CI 0.50, 1.06) BMI unit increase in offspring's BMI, respectively. When including offspring's energy intake (Model 3, Table S4) to the adjusted model, the association between mother's and father's BMI and the offspring's BMI was of same strength.

#### 3.3.2. Associations Between Parents' and Offspring's Waist Circumference

In the subsample of 315 parent-offspring trios, we found a positive association between mother's and offspring's waist circumference. One SD increase in mother's waist circumference (11 cm) was associated with 3.07 (95% CI 1.67, 4.49) cm increase in offspring's waist circumference when adjusted for all covariates (Model 2, Table 3). No interaction was found between mother's waist circumference and offspring's sex (*p*=0.38). No significant association was seen between father's and offspring's waist circumference (1.10 [95% CI −0.31, 2.51] in the fully adjusted model [Model 2, Table 3]).

## 4. Discussion

### 4.1. Main Findings

#### 4.1.1. BMI and Obesity Status

We found strong associations in BMI and obesity status at middle age between parents (aged 40–59 years in 1994–1995) and their adult offspring (aged 40–59 years in 2015–2016). Associations remained strong after adjusting for offspring's and parents' education level, offspring's physical activity level, and total energy intake, indicating that the associations are not explained by these variables. Our findings are in accordance with previous research in children and adolescents [7, 28], young adults [9, 10], and adults of all ages [12, 15]. Likewise, the increased risk of obesity if one parent had obesity, and higher risk if both parents had obesity, is found in studies of children and adolescents [27, 29] and young adults [10]. Obesity in childhood and adolescence rarely reverses but follows the individual into adulthood [30]. It is, however, found that individuals who reverse childhood and adolescence obesity and become adults without obesity have a similar cardiovascular risk profile as those who never had obesity [31]. Previous studies have demonstrated that the parents-offspring associations in BMI and obesity persist into young adulthood [9, 10], and our findings indicate that these associations remain evident also in middle age.

#### 4.1.2. Sex Differences

We observed a tendency that the association with mother's BMI and obesity status was stronger for daughters compared to sons. However, test of interaction between parents' BMI and offspring's sex, as well as the overall interaction between parents' obesity status and offspring's sex, were not statistically significant.

Studies in children and adolescents show conflicting results, where one study showed stronger associations in overweight between a child and the parent of the same sex, i.e., mother-daughter and father-son relationships in 5–8 -year-old [18], one showed that overweight in daughters is associated with only maternal overweight in 11-year-old [32], and one study showed no significant difference between sexes (age 13–19 years) [29]. Similar results are found in adult offsprings where some find stronger associations between parent and offspring of the same sex [13], some find stronger mother-daughter compared to mother-son (and equal parent-son association) [12, 33], and some find no sex difference [14]. Despite some conflicting conclusions (possibly due to differences in sample sizes, age, and statistical models), together they point toward a stronger association between mother-daughter compared to mother-son relationships [12]. This is in accordance with our borderline significant sex differences indicating that mother's BMI and obesity status may play a more important role when it comes to daughter's BMI and obesity status compared to their son's, although both parents' BMI are associated with both daughter's and son's BMI. Others have discussed that this trend could be explained by the mother acting as the primary caregiver of adolescents, thus having a stronger influence [32]. Mothers may be more involved in meal preparation and are an important role model during mealtime, especially during the first years, and may therefore influence eating behaviors more strongly [34]. Energy intake in offsprings, especially daughters, is found to be associated with mother's, but not father's energy intake [35]. In terms of behavior in general, the parent of the same sex may act as the most important role model for their children, explaining the sex-assortative association in BMI observed in some studies [18]. Perez-Pastor et al. [18] conclude that the observed sex-differences in their study are less likely to be due to genes and thus more environmentally rooted. This could explain why the sex differences are not as strong at age 40–59 as during childhood and adolescence. To still see traces of this sex-difference in middle-aged offsprings, clearly shows the significant role of our familial background in relation to obesity.

We found positive association between mother's and offspring's waist circumference, but no significant interaction with offspring's sex. There was no significant association between father's and offspring's waist circumference. Other studies of children/adolescent offsprings find positive associations with both parents when not stratifying on offspring's sex [29] and a mother-daughter father/mother-son relationship when stratifying on offspring's sex [32]. The lack of association in waist circumference between father and offspring in our study could be due to the subsample being substantially smaller than the main sample. However, this was accounted for by including all 315 trios of both daughters and sons in the same model to achieve more power (instead of stratifying on offspring's sex). Our findings indicate that mother's waist circumference is more strongly associated with offspring's waist circumference, as in accordance with our main results, as well as other studies finding a stronger effect from maternal BMI on offspring's BMI.

#### 4.1.3. Family Matters

The findings of this study highlight the significant role of family history in the persistence of obesity into middle-aged adulthood. The strong associations observed between parents' and offspring's BMI and obesity status, even after adjusting for relevant potential confounding or mediating lifestyle factors, show that obesity is deeply rooted in family lines. The persistence of obesity across generations suggests that obesity should be approached not just as an individual health issue but as a familial and a community health concern. Early prevention and intervention strategies are required to ease the long-term somatic and psychological effects [2] of living a lifetime with obesity. Additionally, these findings highlight the role of maternal BMI and waist circumference in relation to offspring's, especially daughter's, BMI and waist circumference, suggesting that interventions could consider these family patterns to more effectively mitigate the effect of obesity passed on in families.

### 4.2. Strengths and Limitations

The Tromsø Study spans over 50 years and thus enables studies using family linkage, which allowed us to include full parent-offspring trios in a specific age range to study familial transmission during a time period when obesity became a world-wide epidemic. The Tromsø Study has had generally high attendance (77% and 65% for Tromsø4 and Tromsø7, respectively), which provides representative data with generalizability to other Scandinavian and North European populations. However, as in other population studies, nonattenders in Tromsø7 have been shown to differ from attenders in terms of sex, age, and several variables related to socioeconomic status [36]. The possibility to use data from parents measured 20 years before the offspring is strength because it adds temporality to otherwise cross-sectional analyses. During these years, there has been a well-known increase in obesity globally [1]. If the increase in obesity prevalence has been larger among those with a genetic predisposition [6], this could possibly strengthen our estimates.

We chose to adjust for offspring and not parents' lifestyle factors (except education) under the assumption that an effect of parents' lifestyle is captured in offspring's lifestyle, i.e., parents' physical activity only affects offspring's BMI through the offspring's own physical activity. This also applies to energy intake.

While several previous studies have relied on self-reported height and weight, our study includes height, weight, and waist circumference measured by trained personnel using standard methods, reducing the risk of misclassification [16]. Additionally, validated questionnaires were used in Tromsø7 for collecting data on diet [25, 26] and physical activity level [24, 37]. Further, self-reported educational level in Tromsø7 has been found to be complete and valid when compared to national registry data [38]. Although anthropometric measurements such as BMI and waist circumference are widely accepted measurements of obesity at the populational level, they cannot distinguish between fat and lean mass, as opposed to body composition measurements. However, previous analyses from Tromsø7 show strong correlations between gram of visceral adipose tissue as assessed by dual X-ray absorptiometry (DXA) and anthropometric measures like BMI and waist circumference, with correlation coefficients of ≥ 0.77 and ≥ 0.80, respectively [39].

## 5. Conclusion

The intergenerational transmission of obesity continues into middle-aged adulthood in this population-based study. We found strong associations between BMI and obesity status in parents and adult offspring, both measured at middle age, 20 years apart. There was a tendency of a stronger association in mother-daughter relationships compared to mother-son. Associations remained strong when adjusting for education level, physical activity level, and energy intake. These findings highlight the importance of early preventive measures to mitigate the long-term health consequences of familial obesity.

## Figures and Tables

**Table 1:** Study sample characteristics. The Tromsø study 1994–2016.

	Adult offspring (Tromsø7 2015–2016)		Parents (Tromsø4 1994–1995)	
	Daughters (*n* = 1028)	Sons (*n* = 1040)	Mothers (*n* = 2068)	Fathers (*n* = 2068)
Age, mean (SD)	46.2 (4.2)	46.3 (4.1)	49.4 (4.5)	51.9 (4.5)
BMI, mean (SD)	26.9 (5.1)	28.3 (4.1)	25.2 (4.0)	26.4 (3.1)
Obesity, % (*n*)	23.4 (240)	29.3 (305)	11.6 (240)	12.9 (267)
Physical activity level^1^, % (*n*)				
Sedentary	12.1 (123)	16.6 (171)		
Low	61.0 (620)	42.8 (441)		
Moderate	24.0 (244)	35.3 (364)		
Vigorous	2.9 (27)	5.2 (54)		
Education^2^, % (*n*)				
Primary	11.8 (121)	16.3 (168)	48.2 (996)	38.6 (798)
Secondary	26.5 (271)	39.8 (411)	32.8 (679)	36.6 (756)
Tertiary short	23.3 (239)	20.2 (209)	10.0 (206)	15.5 (320)
Tertiary long	38.4 (393)	23.7 (245)	8.8 (181)	9.2 (191)

*Note:* Missing data on physical activity in offspring (*n* = 22). Missing data on education in offspring (*n* = 11) and education in parents (*n* = 9).

Abbreviations: BMI, body mass index; SD, standard deviation.

^1^Self-reported leisure time physical activity in four categories (coded 1–4).

^2^Self-reported educational level in four categories (coded 1–4).

**Table 2:** Linear regression coefficients for the association between parents' and offspring's body mass index by offspring's sex. The Tromsø study 1994–2016.

	Model 1^1^		Model 2^2^	
	Daughters *N* = 1028	Sons *N* = 1040	Daughters *N* = 1009	Sons *N* = 1021
BMI mother (per 1 SD^3^)	1.03 (0.71, 1.36)	0.63 (0.37, 0.88)	0.95 (0.63, 1.27)	0.62 (0.38, 0.87)
BMI father (per 1 SD^3^)	0.80 (0.47, 1.13)	0.71 (0.46, 0.96)	0.80 (0.48, 1.12)	0.68 (0.43, 0.92)
Physical activity (per level)			−1.57 (−2.0, −1.13)	−0.98 (−1.28, −0.69)
Education offspring (per level)			−0.41 (−0.70, −0.11)	−0.04 (−0.30, 0.21)
Education mother (per level)			−0.19 (−0.58, 0.19)	−0.31 (−0.61, −0.01)
Education father (per level)			−0.13 (−0.51, 0.25)	−0.20 (−0.49, 0.10)

*Note:* Numbers are regression coefficients with 95% confidence intervals from linear mixed models.

Abbreviations: BMI, body mass index; SD, standard deviation.

^1^Adjusted for parents' and offspring's age.

^2^Adjusted for parents' age and education level and offspring's age, physical activity and education level.

^3^One SD corresponds to 4.0 kg/m^2^ and 3.1 kg/m^2^ for mothers and fathers, respectively.

**Table 3:** Linear regression coefficients for the association between parents' and offspring's waist circumference. The Tromsø study 1994–2016.

	Model 1^1^ *N* = 315	Model 2^2^ *N* = 313
Waist circumference mother (per 1 SD^3^)	3.42 (1.93, 4.92)	3.07 (1.67, 4.49)
Waist circumference father (per 1 SD^3^)	0.94 (−0.55, 2.42)	1.10 (−0.31, 2.51)
Physical activity		−4.69 (−6.41, −2.97)
Education offspring (per level)		−0.64 (−1.91, 0.62)
Education mother (per level)		−1.80 (−3.71, 0.11)
Education father (per level)		0.74 (−1.02, 2.50)

*Note:* Numbers are regression coefficients with 95% confidence intervals from linear mixed models.

Abbreviations: BMI, body mass index; SD, standard deviation.

^1^Adjusted for parents' and offspring's sex, age.

^2^Adjusted for parents' age and education level and offspring's sex, age, physical activity and education level.

^3^One SD corresponds to 11.0 cm and 9.5 cm in mothers and fathers, respectively.

## Data Availability

The data underlying this study was provided by the Tromsø Study by permission and is not publicly available, due to legal restrictions on data availability, in order to control for data sharing, including publication of datasets with the potential of reverse identification of de-identified sensitive participant information. The data can, however, be made available upon application to the Tromsø Study (The Tromsø Study, Department of Community Medicine, Faculty of Health Sciences, UiT The Arctic University of Norway, email: tromsous@uit.no) by following the steps presented on The Tromsø Study website: https://uit.no/research/tromsostudy.

## Supporting Information

Table S1: shows linear regression coefficients for the association between parents' and offspring's height by offspring's sex.

Table S2: shows linear regression coefficients for the association between parents' and offspring weight by offspring's sex.

Table S3: shows risk ratios for offspring's obesity status according to obesity status in parents by offspring's sex.

Table S4: shows linear regression coefficients for the association between parents' and offspring's body mass index in a subsample with dietary data.
