# Supplementary material for: Tracing Obesity From Parents to Adult Offspring: The Tromsø Study 1994–2016
Source: J Obes. 2025 Oct 11;2025:8834694. doi: 10.1155/jobe/8834694 (PMC12535470; doi:10.1155/jobe/8834694)
Supplement: Supporting Information — Additional supporting information can be found online in the Supporting Information section. [file 8834694.f1.zip › Supporting information_2.docx]

**Table S1. Linear regression coefficients for the association between parents’ and offspring’s height by offspring’s sex. The Tromsø Study 1994-2016.**

|  | Daughters  n=1009 | Sons  n=1021 |
| --- | --- | --- |
| Height mother (per SD) | 2.99 (2.68, 3.29) | 2.66 (2.33, 2.99) |
| Height father (per SD) | 2.26 (1.95, 2.56) | 2.68 (2.35, 3.01) |
| Physical activity (per level) | 0.40 (-0.03, 0.83) | -0.23 (-0.62. 0.15) |
| Education offspring (per level) | 0.25 (-0.04, 0.54) | 0.05 (-0.43, 0.33) |
| Education mother (per level) | -0.18 (-0.55, 0.18) | -0.05 (-0.43, 0.33) |
| Education father (per level) | -0.16 (-0.52, 0.20) | 0.13 (-0.25, 0.50) |

Numbers are regression coefficients with 95 % confidence intervals from linear mixed models. ^1^Adjusted for parents’ age and education and offspring’s age, physical activity and education.
SD; standard deviation.
^2^One SD corresponds to 5,9 cm and 6,2 cm for mothers and fathers, respectively.

**Table S2. Linear regression coefficients for the association between parents' and offspring weight by offspring’s sex. The Tromsø Study 1994-2016.**

|  | Daughters  n=1009 | Sons  n=1021 |
| --- | --- | --- |
| Weight mother (per SD) | 2.81 (1.84, 3.78) | 2.12 (1.22, 3.02) |
| Weight father (per SD) | 2.71 (1.63, 3.79) | 2.54 (1.56, 3.52) |
| Physical activity (per level) | -3.98 (-5.23, -2.73) | -3.37 (-4.38, -2.36) |
| Education offspring (per level) | -0.87 (-1.72, 0.02) | -0.09 (-0.96, 0.78) |
| Education mother (per level) | -0.57 (-1.68, 0.54) | -0.95 (-1.98, 0.09) |
| Education father (per level) | -0.30 (-1.40, 0.80) | -0.38 (-1.40, 0.63) |

Numbers are regression coefficients with 95 % confidence intervals from linear mixed models.
^1^Adjusted for parents’ age, height, and education and offspring’s age, physical activity, and education.
SD; standard deviation.
^2^One SD corresponds to 11,3 kg and 11,5 kg for mothers and fathers, respectively.

|  | Model 1^1^ | | | | Model 2^2^ | |
| --- | --- | --- | --- | --- | --- | --- |
|  | Daughters  n=1028 | | Sons  n=1040 | | Daughters  n=1009 | Sons  n=1021 |
|  | No. | RR (95% CI) | No. | RR (95% CI) | RR (95% CI) | RR (95% CI) |
| Obesity^3^ status parents |  |  |  |  |  |  |
| Both normal weight | 229 | 1 (reference) | 207 | 1 (reference) | 1 (reference) | 1 (reference) |
| Overweight^3^ in one or both | 578 | 1.62 (1.12, 2.36) | 582 | 1.35 (1.00, 1.84) | 1.69 (1.17, 2.45) | 1.43 (1.05, 1.94) |
| Obesity in mother | 93 | 3.48 (2.31, 5.22) | 112 | 1.75 (1.20, 2.56) | 3.29 (2.19, 4.93) | 1.81 (1.24, 2.64) |
| Obesity in father | 109 | 2.86 (1.87, 4.37) | 123 | 2.33 (1.66, 3.26) | 2.70 (1.76, 4.15) | 2.30 (1.65, 3.20) |
| Obesity in both | 19 | 3.59 (1.86, 6.82) | 16 | 2.79 (1.64, 4.72) | 3.36 (1.73, 6.52) | 3.01 (1.71, 5.30) |
| Physical activity (per level) |  |  |  |  | 0.64 (0.55, 0.76) | 0.69 (0.61, 0.77) |
| Education offspring (per level) |  |  |  |  | 0.91 (0.82, 1.01) | 1.02 (0.92, 1.14) |
| Education mother (per level) |  |  |  |  | 0.88 (0.76, 1.03) | 0.83 (0.73, 0.96) |
| Education father (per level) |  |  |  |  | 0.98 (0.85, 1.13) | 0. 98 (0.87, 1.11) |

**Table S3. Risk ratios of offspring’s obesity status according to obesity status in parents by offspring's sex. The Tromsø Study 1994-2016.**

Risk ratios with 95 % confidence intervals from generalized estimating equation models. ^1^Adjusted for parents’ and offspring’s age.
^2^Adjusted for parents’ age and education and offspring’s age, physical activity and education.

^3^Overweight defined as BMI 25.0-29.9 kg/m^2^ and obesity as BMI ≥30.0 kg/m².
BMI; body mass index, No.; Number of subjects, RR; risk ratio

**Table S4. Linear regression coefficients for the association between parents’ and offspring’s body mass index in a subsample with dietary data. The Tromsø Study 1994-2016.**

|  | Model 1^1^  N=1065 | Model 2^2^  N=1055 | Model 3^3^ N=1055 |
| --- | --- | --- | --- |
| BMI mother (per 1 SD^4^) | 0.83 (0.54, 1.12) | 0.75 (0.47, 1.03) | 0.74 (0.46, 1.02) |
| BMI father (per 1 SD^4^) | 0.83 (0.54, 1.12) | 0.78 (0.50, 1.06) | 0.78 (0.50, 1.05) |
| Physical activity |  | -1.40 (-1.75, -1.04) | -1.44 (-1.80, -1.09) |
| Education offspring (per level) |  | -0.51 (-0.80, -0.23) | -0.51 (-0.79, -0.23) |
| Education mother (per level) |  | -0.05 (-0.39, 0.28) | -0.05 (-0.39, 0.28) |
| Education father (per level) |  | -0.16 (-0.49, 0.18) | -0.16 (-0.49, 0.18) |
| Energy intake (per MJ) |  |  | 0.10 (0.01, 0.19) |

Numbers are regression coefficients with 95 % confidence intervals from linear mixed models.

^1^Adjusted for parents’ and offspring’s sex, age.
^2^Adjusted for parents’ age and education level and offspring’s sex, age, physical activity and education level.
^3^Adjusted for parents’ age and education level and offspring’s sex, age, physical activity and education level, energy intake.
^4^One SD corresponds to 4.00 kg/m^2^ and 3.00 kg/m^2^ in mothers and fathers, respectively
BMI; body mass index, SD; standard deviation.
